# Influence of *PPM1D* Mutations on Response and Survival Outcomes Following Bispecific Antibody Therapy in Relapsed and Refractory Multiple Myeloma Patients

**DOI:** 10.3390/biomedicines14061392

**Published:** 2026-06-20

**Authors:** Elena Fiori, Martina Bertschinger, Ulrike Bacher, Michele Hoffmann, Henning Nilius, Katja Seipel, Thomas Pabst

**Affiliations:** 1 Department of Medical Oncology, Inselspital, Bern University Hospital, 3010 Bern, Switzerland; elena.fiori@students.unibe.ch (E.F.); martina.bertschinger@insel.ch (M.B.); michele.hoffmann@insel.ch (M.H.); 2 Department of Hematology, Inselspital, Bern University Hospital, 3010 Bern, Switzerland; veraulrike.bacher@insel.ch; 3 Department for Biomedical Research (DBMR), University of Bern, 3008 Bern, Switzerland; katja.seipel@unibe.ch; 4 Department of Clinical Chemistry, Inselspital—Bern University Hospital, 3010 Bern, Switzerland; henning.nilius@insel.ch

**Keywords:** multiple myeloma, bispecific antibodies, *PPM1D* mutations, clonal hematopoiesis

## Abstract

**Background/Objectives:** Therapeutic options for patients with relapsed and refractory multiple myeloma (RRMM) have advanced substantially in recent years. In particular, T-cell-engaging therapies, including chimeric antigen receptor (CAR) T-cell therapy and bispecific antibodies (bsAbs), have emerged as highly effective treatment modalities. However, data on predictive biomarkers for response to these therapies remain limited. Patients currently receiving T-cell-engaging therapies are typically heavily pretreated and frequently exhibit clonal hematopoiesis. Clonal hematopoiesis, especially involving *PPM1D* mutations, may adversely affect the efficacy of T-cell-engaging therapies. **Methods:** We conducted a retrospective, single-center study including 27 patients with RRMM who were treated with bsAbs (teclistamab, elranatamab, or talquetamab) between June 2022 and September 2025 and for whom genetic material was available before bsAB treatment. We evaluated the impact of *PPM1D* mutations on treatment response, progression-free survival (PFS), and overall survival (OS). **Results:** The prevalence of *PPM1D* mutations in our cohort was 27%. Compared with patients without *PPM1D* mutations, mutation carriers showed a trend toward less deep remissions and demonstrated significantly inferior 6-month PFS (43% vs. 85%, *p* = 0.0272) and 6-month OS (57% vs. 90%, *p* = 0.0473). **Conclusions:** These findings suggest that *PPM1D* mutations may represent a promising biomarker in patients with RRMM treated with bsAbs. Larger, prospective studies are warranted to validate and further elucidate these observations.

## 1. Introduction

Multiple myeloma (MM) is a hematologic malignancy characterized by the clonal proliferation and accumulation of malignant plasma cells [1]. Together with the complex interaction between malignant plasma cells and the bone marrow microenvironment, this process gives rise to a heterogeneous clinical phenotype, which commonly includes fatigue, anemia, recurrent infections, bone lesions, hypercalcemia and renal failure [2]. Despite substantial therapeutic advances, MM remains incurable, and disease relapses are inevitable for the majority of patients. Due to the selection of resistant plasma cell clones, with every relapse, treatment becomes increasingly challenging.

Novel immunotherapeutic approaches have emerged for patients with relapsed and refractory MM (RRMM), most notably chimeric antigen receptor (CAR) T-cell therapies and bispecific antibodies (BsAbs). These approaches lead to potent immune activation, modulation and a previously unseen efficacy in RRMM [3,4].

BsAbs represent a class of targeted, off-the-shelf immunotherapy methods where antibodies simultaneously bind to a surface antigen on the malignant plasma cell and to a surface antigen on an immune effector cell, typically CD3 on T-cells [5]. This dual engagement enhances immune synapse formation, amplifies T-cell-mediated cytotoxicity, and promotes efficient lysis of malignant plasma cells, including those with low target antigen expression [3]. The most advanced BsAbs in the treatment of RRMM are directed against B-cell maturation antigen (BCMA), elranatamab [6] and teclistamab [7] or against G protein-coupled receptor family C group 5 member D (GPRC5D), such as talquetamab [8].

Cytotoxic therapies, including conventional chemotherapy, may confer a selective advantage to mutant hematopoietic stem cell (HSC) populations under conditions of genotoxicity [9]. This selective pressure, along with the prolonged survival of these hematopoietic cell clones, may facilitate the accumulation of additional mutations [10]. Progressive clonal expansion of such mutant clones can culminate in secondary hematologic malignancies. This process, characterized by the expansion of distinct hematopoietic clones, is known as clonal hematopoiesis (CH) [9,11]. The genes most frequently implicated in CH include *PPM1D*, *TP53*, *DNMT3A*, *ASXL1* and *TET2* [11,12,13]. *PPM1D,* located on chromosome 17q23 [14], encodes protein phosphatase Mg^2+^/Mn^2+^-dependent 1D (PPM1D, Wip1), a serine–threonine phosphatase that is transcriptionally activated by p53. *PPM1D* negatively regulates the DNA damage response pathway through a feedback mechanism, thereby attenuating apoptotic signaling [9,10,11,15]. *PPM1D* is expressed in HSC as well as in mature cells, including B- and T-lymphocytes [11,16]. *PPM1D* mutations are typically located on the terminal region of exon 6, resulting in C-terminal truncation and impaired proteasomal degradation of the protein [10,15].

The consequent accumulation of the *PPM1D* protein enhances suppression of apoptosis, thus promoting cellular survival.

As MM is an uncurable disease and bsAbs are only approved in further line treatment; most patients who receive bsAB treatment have as of today undergone multiple prior lines of therapy, including chemotherapy, before the bsAB treatment. Previous studies could demonstrate a correlation of prior exposure of cytotoxic agents and radiotherapy with *PPM1D* mutations, which is explained by a clonal selection of *PPM1D* mutant cells [17,18,19]. Thus, CH is frequently detected in patients with RRMM [20,21].

Given the increased prevalence of *PPM1D* mutations in patients previously exposed to cytotoxic therapies, this retrospective study investigates the correlation between these mutations and clinical outcomes following BsAb therapy in patients with RRMM.

## 2. Materials and Methods

### 2.1. Patient Population

In this retrospective single-center study, conducted at Inselspital, University Hospital of Bern, we investigated patients with RRMM who were treated with different BsAbs, including teclistamab and elranatamab (targeting BCMA) and talquetamab (targeting GPRC5D), between 7 June 2022 and 1 September 2025. A precondition for the study was the availability of genetic material to determine the *PPM1D* gene status before initiation of bsAB treatment. The study was conducted according to the guidelines of the Declaration of Helsinki and approved by the Ethics Committee of the Canton of Bern, Switzerland (decision number 2024-01189 and date of approval of 25 June 2024). All patients included have given informed consent to participate in the study.

### 2.2. Study Endpoints

The primary endpoints were the 6-month overall survival (OS) and the 6-month progression-free survival (PFS). PFS was defined as the time from initiation of bsAB therapy to disease progression or death from any cause. OS was defined as the time from initiation of bsAb therapy to death from any cause. As secondary endpoints, median PFS and OS were assessed. PFS and OS were censored on 1 September 2025, which served as the cutoff date.

### 2.3. Patient Characteristics and Therapy Response

Clinical parameters included demographic data, laboratory parameters, and MM-specific characteristics, such as R-ISS stage and cytogenetic risk. Data regarding the disease status prior to BsAb therapy, as well as the number and types of prior treatments and responses, were collected. Regarding the BsAb treatment, information about the administered agent, the treatment duration and clinical outcome was recorded. Outcomes included the best observed response, as well as adverse events, such as infections, cytokine release syndrome (CRS), immune effector cell-associated neurotoxicity syndrome (ICANS), and other treatment-related toxicities. The best observed response was complete remission (CR), followed by very good partial response (VGPR) and partial response (PR). Stable disease (SD) was defined as not meeting the criteria for CR, VGPR, PR and progressive disease (PD). Negative responses were identified as PD or death.

It was not possible to retrieve complete clinical information for all patients. In cases of missing data, the corresponding variables were recorded as “no information” (no info).

### 2.4. PPM1D Gene Analysis

Peripheral blood samples obtained from patients and collected within the framework of the institutional biobank were used for the analysis. Assessment of *PPM1D* status was performed before the start of bsAB therapy. Genomic DNA was extracted from peripheral blood mononuclear cells of 27 patients. Mutations in exon 6 of the *PPM1D* gene were identified using an amplicon-based next-generation sequencing (NGS) approach, as previously described [12].

### 2.5. Data Collection

Clinical data were collected from the Epic electronic health record system. Statistical analyses and the generation of figures were performed using GraphPad Prism 10.0. *p*-values were calculated using Fisher’s exact test or the chi-square test. Values below 0.05 were considered statistically significant.

## 3. Results

### 3.1. Prevalence of PPM1D Mutations

Through an amplicon-based NGS approach, as previously described by Vellky et al. and Fandrei et al. [22,23], we identified seven out of 27 patients carrying mutations in exon 6 of *PPM1D* gene, corresponding to a prevalence of 26%. Based on these results, the whole cohort was stratified into two groups: the *PPM1D*-mutated (mut) and the *PPM1D* wild-type (wt) groups. The VAF of *PPM1D* mutations detected in PBMC-derived DNA samples ranged from 0.011 to 0.018 (Table S1, Supplementary Materials).

### 3.2. Patient Characteristics

Data from 27 patients who met the inclusion criteria were retrospectively included. Baseline characteristics are summarized in Table 1. Regarding sex distribution, the *PPM1D*-mut group was predominantly male (71%). At the initial diagnosis of MM, the median age of the entire cohort was 60.4 years. The *PPM1D*-mut group showed a slightly higher median age (65.6 years) compared to the *PPM1D*-wt group (58 years).

The majority of patients from the whole cohort had R-ISS stage II (33%). Cytogenetic high-risk included the presence of t(4;14), t(14;16), t(14;20), gain/amp(1q21), del(1p) and del(17p) mutations. These genetic mutations were found in 44% of patients during initial diagnosis.

The whole cohort was heavily pretreated. A total of 74% of patients in the overall cohort had at least 3–4 lines of previous anti-myeloma-directed therapies. In the *PPM1D*-wt group 70% of patients had received ≥3 prior lines of therapy, whereas 86% of patients in the *PPM1D*-mut group had undergone more than two previous lines of therapy. More than half of the patients (63%) in the overall cohort had undergone high-dose chemotherapy followed by autologous hematopoietic stem cell transplantation (HDCT/ASCT), including 43% in the *PPM1D*-mut group and 70% in the *PPM1D*-wt group.

Before initiating BsAb therapy, 41% of patients in the entire cohort had previously received a BCMA-directed CAR T-cell therapy, including 14% in the *PPM1D*-mut group and 50% in the *PPM1D*-wt group (Table 1).

### 3.3. Bispecific Antibodies

In the overall cohort, a total of 31 patients received BsAb therapy. Four patients, two in each *PPM1D* subgroup (mutated and wild type), received more than one agent (talquetamab and teclistamab). Overall, 42% of the patients received talquetamab, 52% teclistamab and 6% elranatamab, with no major differences in treatment distribution between the *PPM1D*-mut group (33%, 56%, 11%) and the *PPM1D*-wt group (45%, 50%, 5%) (Table 2).

The median age at the initiation of BsAb treatment was 67.6 years in the overall cohort, compared with 73.7 years in the patients harboring *PPM1D*-mut and 65.4 years in the *PPM1D*-wt group. Treatment-related adverse events were common, and only 7% of the patients did not experience any toxicities. The most frequently observed toxicities comprised CRS, infections, thrombocytopenia, dysgeusia and cutaneous toxicities. ICANS was rare and occurred in a single patient, who belonged to the *PPM1D*-mut group.

Before undergoing BsAb therapy, most patients (78%) had progressive disease (PD), with comparable rates in the *PPM1D*-mut group (78%) and the *PPM1D*-wt group (82%).

Regarding treatment duration, a wide variability was observed. Overall, 16% of patients received less than one full cycle of BsAb therapy, including 33% of patients in the *PPM1D*-mut group and 9% in the *PPM1D*-wt group. Most patients (35%) received between one and five treatment cycles, comprising 22% of the PPM1D-mut and 41% of the *PPM1D*-wt cohort. Notably, 19% of all patients received prolonged BsAb therapy exceeding ten cycles, with comparable proportions in the *PPM1D*-mutant (22%) and *PPM1D*-wt (18%) groups.

### 3.4. Clinical Outcomes

The best treatment responses differed between the two subgroups. CR was more frequently seen in the *PPM1D*-wt group (41%) compared to the *PPM1D*-mut group (11%) (*p* = 0.0543). In contrast, PD was the most common outcome in the *PPM1D*-mut cohort (44%), compared with 18% in the *PPM1D*-wt cohort. Death occurred in 33% in the *PPM1D*-mut cohort, while only one patient died in the *PPM1D*-wt group (5%) (Table 3).

Survival outcomes following initiation of BsAb therapy differed significantly between patients harboring the *PPM1D* mutation.

**Figure 1:**
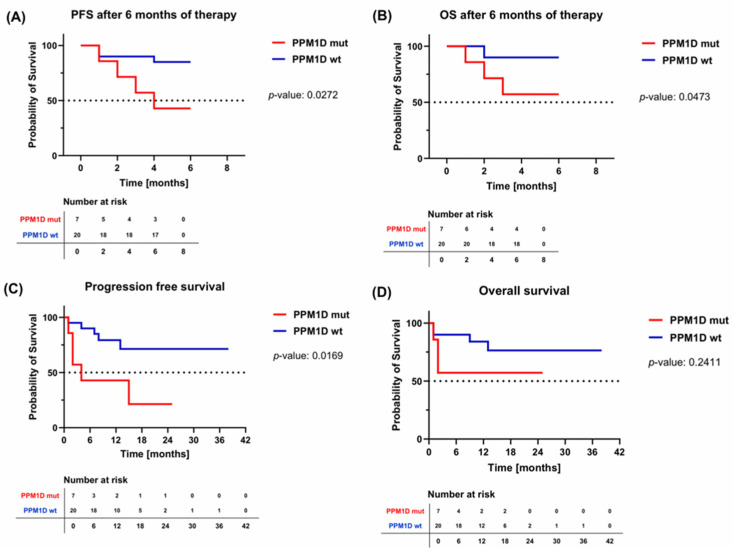
PFS and OS in MM patients treated with BsAbs analyzed by Kaplan–Meyer and stratified by *PPM1D* status: PFS after 6 months of BsAB therapy (**A**), OS after 6 months of therapy (**B**), progression-free survival (**C**) and overall survival (**D**).

The 6-month PFS was significantly lower in the *PPM1D*-mut cohort (43%) compared with the *PPM1D*-wt group (85%), (*p* = 0.0272; Figure 1A). Similarly, the 6-month OS was significantly lower in the *PPM1D*-mut cohort (57%) compared with the *PPM1D*-wt group (90%) (*p* = 0.0473; Figure 1B).

The median PFS was 4 months in the *PPM1D*-mut cohort, whereas it was not reached in the *PPM1D*-wt subgroup (*p* = 0.0169; Figure 1C). The median OS was not reached in either cohort (*p* = 0.2411; Figure 1D).

Additionally, a multivariate analysis was performed to adjust for risk factors. *PPM1D* mutations emerged as an independent adverse prognostic factor for PFS (HR 4.01, *p* = 0.022), indicating a significantly increased risk of disease progression. A similar trend was observed for OS but did not reach statistical significance (HR 4.13, *p* = 0.084) (Table 4).

## 4. Discussion

In this retrospective study, we investigated the prevalence and the association of *PPM1D* mutations on clinical outcomes in RRMM patients following BsAb therapy. Among the 27 patients analyzed we detected presence of *PPM1D* mutation in 26% (7/27) of the patients. In previous studies, we reported comparable prevalences of *PPM1D* mutations in other hematological malignancies, with rates of 25% in mantle cell lymphoma (MCL) and 20.5% in diffuse large B-cell lymphoma (DLBCL), whereas a lower prevalence was observed in MM patients (5.3%) [11,12,24].

Baseline patient characteristics were generally comparable between the two groups. However, patients in the *PPM1D*-mut cohort were older during diagnosis than those in the *PPM1D*-wt group (median age, 65.6 vs. 58 years; *p* = 0.0808). Similarly, R-ISS stage and cytogenetic risk tended to be higher among *PPM1D*-mut patients, but not statistically significant. Overall, there was a slight male predominance. All patients included in this study had received multiple prior lines of therapy before BsAb treatment. Patients in the *PPM1D*-mut group had undergone a higher number of previous treatments. This finding is consistent with our previous data in MM patients showing an increased prevalence of *PPM1D* mutations after multiple lines of therapy [12]. Regarding disease status at the beginning of BsAb therapy, no differences were observed between the two groups, as the majority of patients presented with PD. Similar to age during diagnosis, patients in the *PPM1D*-mut cohort were older at the time of treatment initiation than those in the *PPM1D*-wt group (median age, 73.7 vs. 65.4 years; *p* = 0.0638). As suggested by Stelmach et al., the prevalence of *PPM1D* mutations increases with age, and older patients are also more likely to have undergone multiple cytotoxic therapies, which may further contribute to the accumulation of this mutation [9,25].

The administration of the different BsAb agents (talquetamab, teclistamab, and elranatamab) did not differ between the two groups, although elranatamab was administered in only two patients. Additionally, two patients in each subgroup received more than one BsAb agent (talquetamab and teclistamab). Similarly, the number of administered BsAb treatment cycles did not differ between the two groups, with a wide range of 1–10 cycles in both *PPM1D* groups.

The incidence of side effects during BsAb therapy did not differ significantly between the cohorts (*p* = 0.7443). CRS and other side effects, such as thrombocytopenia and dysgeusia, were the most frequently observed toxicities, occurring in 30% and 33% of the overall cohort. Notably, ICANS was reported in only one patient, and this patient belonged to the *PPM1D*-mut group. However, larger cohorts are required to draw conclusions regarding treatment-related adverse effects.

In the overall study population, 32% of the patients achieved CR, with rates of 11% in the *PPM1D*-mut group and 41% in the *PPM1D*-wt group (*p* = 0.0543). PD was more frequently documented in the *PPM1D*-mut patients (44%) in comparison to the *PPM1D*-wt group (18%). These findings are consistent with the expected association of *PPM1D* mutations and poorer outcomes. In addition, death before the cutoff date was observed in 33% of the *PPM1D*-mut cohort, compared with 5% in the *PPM1D*-wt patient group. Furthermore, other studies have reported that CH-associated mutations, such as those in *PPM1D* or *DNMT3A*, have been associated with poorer outcomes following therapies including HDCT/ASCT and CAR T-cell therapy in patients with diverse hematological malignancies [10,12]. Additionally, Awada et al. reported that CH-derived therapy-related myeloid neoplasms (tMN) tended to have worse outcomes after HDCT/ASCT compared with non-CH-derived tMN [26].

The median PFS in the *PPM1D*-mut group was 4 months and varied significantly compared to the PFS in the *PPM1D*-wt group which was not reached (*p* = 0.0169). These results are consistent with a potential association between *PPM1D* mutations and poorer outcomes, as patients with RRMM carrying *PPM1D* mutations may experience earlier relapses after BsAb therapy compared with wild-type patients. Although the median OS was not reached in any cohort (*p* = 0.2411), the survival curve (Figure 1D) shows a trend toward poorer survival in the *PPM1D*-mut group.

Our primary endpoints, PFS and OS at 6 months following BsAb therapy initiation, differed significantly between the two groups. In the overall cohort, the 6-month PFS was 74%. When stratified by *PPM1D* status, the 6-month PFS was 85% in the *PPM1D*-wt group and 43% in the *PPM1D*-mut group (*p* = 0.0272). Similarly, the 6-month OS differed significantly between the two groups with a much higher OS rate in the *PPM1D*-wt group (90%) than in the *PPM1D*-mut group (57%) (*p* = 0.0473).

The mechanism by which *PPM1D* mutations may influence the efficacy of bsAB remains not fully understood. It is well established that *PPM1D* stop-gain or frameshift mutations, which are associated with a loss of the C-terminal degron, lead to an increase in *PPM1D* function. The truncated *PPM1D* protein leads to the inactivation of the tumor suppressor p53, resulting in a lack of negative cell cycle regulation and, consequently, resistance under DNA-damaging conditions. This explains why cells with *PPM1D* mutations respond less effectively to chemotherapy [27]. By promoting cell cycle dysregulation and cell proliferation, the presence of *PPM1D* mutations may impair the optimal response to bsAb therapy. Furthermore, as *PPM1D* mutations occurring in the myeloid compartment can induce a pro-inflammatory state, the immunosuppressive microenvironment may contribute to reduced efficacy of bsAb therapy [28,29].

It should be noted that these findings are based on a retrospective and single-center analysis with a very limited patient number. Furthermore, as previously discussed, it should be noted that the group carrying the *PPM1D* mutations was older than the group without *PPM1D* mutations (73.7 vs. 65.4 years, *p* = 0.0638). However, in a multivariate analysis the *PPM1D* mutation was shown to be an independent risk factor (Table 4), even if the modest sample size limited the precision of our Cox-model estimates.

To prove the effect of *PPM1D* mutations, larger cohorts and prospective trials are warranted to validate these findings and to further characterize the prognostic and predictive relevance of *PPM1D* mutations in patients with RRMM treated with BsAbs.

## 5. Conclusions

Taken together, our findings suggest that *PPM1D* mutations in RRMM patients treated with BsAbs are associated with limited responses to treatment, earlier relapses and inferior short-term survival outcomes. This observation may reflect that *PPM1D* mutations lead to a more aggressive disease biology and/or reduced treatment sensitivity. Independent larger studies will evaluate the meaning of *PPM1D* mutations in RRMM patients treated with BsAbs.

## Figures and Tables

**Table 1:** Patient baseline characteristics at diagnosis of MM.

Parameter	All Patients (*n* = 27)	*PPM1D*-mut (*n* = 7)	*PPM1D*-wt (*n* = 20)	*p*-Value
**Sex**				0.8024
Female	11 (41%)	2 (29%)	9 (45%)	
Male	16 (59%)	5 (71%)	11 (55%)	
**Age at diagnosis, median (years)**	60.4 (41–80)	65.6 (55–80)	58 (41–75)	0.0808
**R-ISS**				0.7549
I	2 (7%)	0 (0%)	2 (10%)	
II	9 (33%)	4 (57%)	5 (25%)	
III	3 (11%)	1 (14%)	2 (10%)	
No info	13 (48%)	2 (29%)	11 (55%)	
**Cytogenetic risk**				0.8947
High	12 (44%)	4 (57%)	8 (40%)	
Standard	4 (15%)	0 (0%)	4 (20%)	
No info	11 (41%)	3 (43%)	8 (40%)	
**Number of prior therapy lines**				0.2417
1–2	3 (11%)	1 (14%)	2 (10%)	
3–4	13 (48%)	2 (29%)	11 (55%)	
5–7	5 (19%)	4 (57%)	1 (5%)	
8–14	2 (7%)	0 (0%)	2 (10%)	
No info	4 (15%)	0 (0%)	4 (20%)	
**Prior HDCT/ASCT**	17 (63%)	3 (43%)	14 (70%)	0.1719
**Prior CAR T-cell therapy**	11 (41%)	1 (14%)	10 (50%)	0.2101

R-ISS: Revised Multiple Myeloma International Staging System, HDCT/ASCT: high-dose chemotherapy/autologous stem cell transplantation, CAR T-cell therapy: chimeric antigen receptor T-cell therapy.

**Table 2:** Bispecific antibody clinical characteristics.

Parameter	All Patients (*n* = 27)	*PPM1D* mut (*n* = 7)	*PPM1D* wt (*n* = 20)	*p*-Value
**BsAb agent**	(*n* = 31 *)	(*n* = 9 *)	(*n* = 22 *)	0.9340
Talquetamab	13 (42%)	3 (33%)	10 (45%)	
Teclistamab	16 (52%)	5 (56%)	11 (50%)	
Elranatamab	2 (6%)	1 (11%)	1 (5%)	
**Age at BsAb therapy, median (years)**	67.6 (42–88)	73.7 (60–88)	65.4 (42–78)	0.0638
**Side effects**				0.7443
Infection	7 (26%)	2 (29%)	5 (25%)	
CRS	8 (30%)	2 (29%)	6 (30%)	
ICANS	1 (4%)	1 (14%)	0 (0%)	
Others	9 (33%)	1 (14%)	8 (40%)	
None	2 (7%)	1 (14%)	1 (5%)	
Disease status before BsAb therapy **	(*n* = 31 *)	(*n* = 9 *)	(*n* = 22 *)	0.6592
CR, VGPR, PR	0 (0%)	0 (0%)	0 (0%)	
SD	1 (3%)	0 (0%)	1 (4%)	
PD	24 (78%)	7 (78%)	18 (82%)	
no info	6 (19%)	2 (22%)	3 (14%)	
**Number of cycles**	(*n* = 31 *)	(*n* = 9 *)	(*n* = 22 *)	0.5186
<1	5 (16%)	3 (33%)	2 (9%)	
1–5	11 (35%)	2 (22%)	9 (41%)	
6–10	5 (16%)	1 (11%)	4 (18%)	
>10	6 (19%)	2 (22%)	4 (18%)	
no info	4 (13%)	1 (11%)	3 (14%)	

* The reported number does not correspond to the number of patients, as some individuals received more than one therapeutic agent. ** The disease status was observed for each BsAb therapeutic agent. Abbreviations; BsAb: bispecific antibody, CRS: cytokine release syndrome, ICANS: immune effector cell-associated neurotoxicity syndrome, CR: complete remission, VGPR: very good partial response, PR: partial response, SD: stable disease, PD: progressive disease.

**Table 3:** Clinical outcomes after BsAb therapy.

Parameter	All Patients (*n* = 31 *)	*PPM1D* mut (*n* = 9 *)	*PPM1D* wt (*n* = 22 *)	*p*-Value
**Best response**				0.0543
CR	10 (32%)	1 (11%)	9 (41%)	
VGPR	4 (13%)	0 (0%)	4 (18%)	
SD	2 (6%)	1 (11%)	1 (5%)	
PD	8 (26%)	4 (44%)	4 (18%)	
Death	4 (13%)	3 (33%)	1 (5%)	
No info	3 (10%)	0 (0%)	3 (13%)	
**Median survival time**				
PFS after 6 months	74%	43%	85%	0.0272
OS after 6 months	81%	57%	90%	0.0473
PFS, months	not reached	4	not reached	0.0169
OS, months	not reached	not reached	not reached	0.2411

* The reported number does not correspond to the number of patients, as some individuals received more than one therapeutic agent. Abbreviations: CR: complete remission, VGPR: very good partial response, SD: stable disease, PD: progressive disease, PFS: progression-free survival, OS: overall survival.

**Table 4:** Multivariable and univariable Cox-proportional hazards models for PFS and OS adjusting for age, sex, HDCT/ASCT status, and CAR-T therapy.

Characteristics	Univariable Regression				Multivariable Regression			
	PFS		OS		PFS		OS	
	HR (95% CI))	*p*-Value	HR (95% CI)	*p*-Value	HR (95% CI)	*p*-Value	HR (95% CI)	*p*-Value
*PPM1D* (yes/no)	3.82 (1.26, 11.6)	0.018	2.79 (0.69, 11.2)	0.15	4.01 (1.22, 13.2)	0.022	4.13 (0.83, 20.7)	0.084
Age	1.47 (0.48, 4.53)	0.5	0.83 (0.21, 3.34)	0.8	0.57 (0.36, 6.76)	0.5	0.45 (0.05, 4.40)	0.5
Sex (male/female)	0.5 (0.16, 1.57)	0.2	0.75 (0.17, 3.33)	0.7	0.57 (0.18, 2.37)	0.5	0.47 (0.07, 3.20)	0.4
HDCT/ASCT (yes/no)	0.6 (0.20, 1.81)	0.4	0.48 (0.12, 1.91)	0.3	0.39 (0.06, 2.67)	0.3	0.02 (0.00, 0.63)	0.026
CAR T (yes/no)	1.3 (0.43, 3.93)	0.6	2.46 (0.58, 10.3)	0.2	3.68 (0.59, 23.1)	0.2	42.6 (2.21, 821)	0.013

Abbreviations: CAR T-cell therapy: chimeric antigen receptor T-cell therapy, CI = Confidence Interval HDCT/ASCT: high-dose chemotherapy/autologous stem cell transplantation, HR = Hazard Ratio.

## Data Availability

Data is available on request due to privacy and ethical restrictions.

## Supplementary Materials

The following supporting information can be downloaded at https://www.mdpi.com/article/10.3390/biomedicines14061392/s1: Table S1. PPM1D gene mutations detected in MM cohort.

## Abbreviations

The following abbreviations are used in this manuscript:

BsAb	Bispecific Antibody
CAR T-cell therapy	Chimeric Antigen Receptor T-Cell Therapy
HDCT/ASCT	High-Dose Chemotherapy/Autologous Stem Cell Transplantation
CRS	Cytokine Release Syndrome
ICANS	Immune Effector Cell-Associated Neurotoxicity Syndrome
CR	Complete Remission
VGPR	Very Good Partial Response
PR	Partial Response
SD	Stable Disease
PD	Progressive Disease
PFS	Progression-Free Survival
OS	Overall Survival
CI	Confidence Interval
HR	Hazard Ratio
