# Supplementary material for: Influence of PPM1D Mutations on Response and Survival Outcomes Following Bispecific Antibody Therapy in Relapsed and Refractory Multiple Myeloma Patients
Source: Biomedicines. 2026 Jun 20;14(6):1392. doi: 10.3390/biomedicines14061392 (PMC13297521; doi:10.3390/biomedicines14061392)
Supplement: Supplementary file 1 [file biomedicines-14-01392-s001.zip › biomedicines-4355766-supplementary.pdf]

Table S1: *PPM1D* mutations

	**AA Change**
0.016	Q510*
0.011	E450*
0.013	Q520*
0.012	L468*
0.018	S484*
0.013	L450*
0.015	E538*
0.014	E475*
0.012	A481fs*
0.015	R552*
0.012	D470fs*
0.011	R458*
0.014	Q510*

Table S1: *PPM1D* gene mutations detected in MM cohort.
